# A novel predictive model incorporating immune-related gene signatures for overall survival in melanoma patients

**DOI:** 10.1038/s41598-020-69330-2

**Published:** 2020-07-27

**Authors:** Mengting Liao, Furong Zeng, Yao Li, Qian Gao, Mingzhu Yin, Guangtong Deng, Xiang Chen

**Affiliations:** 1 Health Management Center, Xiangya Hospital, Central South University, Changsha, 410008 China; 2 Department of Dermatology, Xiangya Hospital, Central South University, Xiangya Road 87, Changsha, 410008 China; 3 Hunan Key Laboratory of Skin Cancer and Psoriasis, Changsha, 410008 China; 4 Hunan Engineering Research Center of Skin Health and Disease, Changsha, 410008 China

**Keywords:** Cancer, Computational biology and bioinformatics, Immunology, Biomarkers

## Abstract

Melanoma is the most invasive type of skin cancer, in which the immune system plays a vital role. In this study, we aimed to establish a prognostic prediction nomogram for melanoma patients that incorporates immune-related genes (IRGs). Ninety-seven differentially expressed IRGs between melanoma and normal skin were screened using gene expression omnibus database (GEO). Among these IRGs, a two-gene signature consisting of CCL8 and DEFB1 was found to be closely associated with patient prognosis using the cancer genome atlas (TCGA) database. Survival analysis verified that the IRGs score based on the signature gene expressions efficiently distinguished between high- and low-risk patients, and was identified to be an independent prognostic factor. A nomogram integrating the IRGs score, age and TNM stage was established to predict individual prognosis for melanoma. The prognostic performance was validated by the TCGA/GEO-based concordance indices and calibration plots. The area under the curve demonstrated that the nomogram was superior than the conventional staging system, which was confirmed by the decision curve analysis. Overall, we developed and validated a nomogram for prognosis prediction in melanoma based on IRGs signatures and clinical parameters, which could be valuable for decision making in the clinic.

## Introduction

Melanoma is an aggressive malignancy with incidence rate constantly growing over the past 40 years^1–3^. There were approximately 287,723 new cases and 60,712 deaths of melanoma in 2018 globally^4^. The prognosis of melanoma is generally unfavorable, with a 5-year overall survival (OS) ranging from 30 to 55% in recent clinical trials for immunotherapy^5,6^. Much effort has been put into identifying biomarkers to evaluate the prognosis of melanoma patients^7,8^. Currently well-established markers include clinicopathologic features such as depth of tumor and ulceration, molecular biomarkers S100, HMB-45 and serum LDH according to the American Joint Committee on Cancer (AJCC) staging system^9–12^. However, these markers still remain deficient given that patients at the same stage could have varied survival outcomes. Therefore, developing more superior biomarkers for melanoma is in urgent need.

Immune system is largely involved in surveillance and elimination of melanoma, while immunosuppression potentiates its proliferation and metastasis^13^. Over the past decade, mechanistic understanding of immune regulation in tumor fueled the development of novel immunotherapy, including checkpoint inhibitors PD-1 (programmed death-1) and CTLA-4 (cytotoxic T-lymphocyte antigen 4) monoclonal antibodies, which transformed the prognosis for many patients^14–17^. Consequently, increasing research began to focus on finding immune-related biomarkers^18^. Typically, PD-L1 expression, tumor mutational burden and tumor infiltrating T cells are shown to be predictive of patient outcomes, but these markers are used only for reference in the clinic due to their insufficient sensitivity and specificity^19–23^. Moreover, the development of combined immune markers recently arose, for single biomarkers can be inadequate to achieve desirable efficiency^24,25^. Despite all these, no combined immune markers are formally validated or recommended as a clinical tool for prognosis^9^.

Bioinformatics analysis based on public database has been used to investigate the prognostic markers in various cancers, with which predictive models can be established to assess individual survival^26^. Until now, there have been a few nomograms for melanoma prognosis prediction, which however, are limited by including merely clinical features as their evaluation indicators, without regard to any gene expression information, let alone combined immune markers^27,28^. In this study, we identified immune-related genes (IRGs) that optimally predicted OS in melanoma, with the use of Gene Expression Omnibus (GEO) and The Cancer Genome Atlas (TCGA) database. For the first time, a prognostic nomogram combining IRGs score with clinical characteristics was constructed, thus providing values for recognizing high risk patients and helping with individualized treatment strategy options.

## Methods

### Data retrieval and processing

Transcriptome profiling datasets and clinical parameters were downloaded from Gene Expression Omnibus (GEO) (https://www.ncbi.nlm.nih.gov/geo) and The Cancer Genome Atlas (TCGA) database (https://xenabrowser.net/datapages). GSE15605 and GSE46517 were used to screen differentially expressed genes (DEGs). TCGA melanoma dataset was selected as the training dataset which included 460 melanoma samples. GSE54467 dataset including 79 melanoma samples was selected as the GEO validation dataset. The DEGs in TCGA and GEO dataset were overlapped and their expressions were normalized using “limma” and “sva” packages in R version 3.6.0 software. IRGs were acquired from the ImmPort database (https://www.immport.org).

### Identification of differential expressed immune-related genes

GSE15605 including 46 primary melanoma patient samples and 16 normal skin samples and GSE46517 consisting of 31 primary melanoma patient samples and 7 normal skin samples were used to extract DEGs using GEO2R. Benjamini & Hochberg false discovery rate method was used as a *P* value adjustment. Adjusted *P* < 0.05 and log (fold change) ≥ 1 were considered as statistically significant. The overlap of the DEGs and IRGs was selected as the set of the differentially expressed immune-related genes (DE-IRGs) for further analysis and visualized via Venn diagram.

### Functional analysis with DE-IRGs

Kyoto Encyclopedia of Genes and Genomes (KEGG) and Gene Ontology (GO) enrichment pathway analyses were performed to investigate the molecular functions, cellular component and biological processes of DE-IRGs. Signaling pathways that significantly related (*P* < 0.05) to DE-IRGs were identified by DAVID (https://david.ncifcrf.gov/). Protein–protein interactions (PPI) network of the DE-IRGs were explore by The STRING database (https://string-db.org) where confidence score ≥ 0.4 was used^29^, and visualized with Cytoscape v. 3.7.1 (https://cytoscape.org/). Hub nodes were identified with the Cytoscape plugin cytoHubba by the maximal clique centrality method. DE-IRGs clusters that strongly correlated in the PPI network were identified with the Cytoscape plugin MCODE. GO enrichment analysis were further performed on DE-IRGs clusters.

### Identification and validation of the prognostic IRGs score

Univariate Cox analysis was first performed to screen the DE-IRGs significantly associated with overall survival (OS) in TCGA melanoma dataset using the “survival” package. Next, genes with *P* < 0.01 by the univariate analysis were chosen for least absolute shrinkage and selection operator (LASSO) logistic regression. Genes with nonzero coefficients were subsequently selected for multivariate Cox analysis to identify the independent prognostic genes. *P* < 0.05 was regarded as statistically significant in the multivariate Cox analysis. With these independent prediction genes, the IRGs score for OS was further calculated as follows: IRG score = β1*X1 + β1*X1 + … + βn*Xn (β: the coefficient derived from multivariate regression; X: gene expression value). The median risk score was chosen as a cutoff value in TCGA melanoma dataset, which was also used to separate patients in GEO validation datasets into high-risk or low-risk group^30–32^. Accordingly, a Kaplan–Meier survival curve was constructed to describe the survival of patients in the high-risk and low-risk group. Furthermore, patient clinicopathological features including age, gender, local ulceration, Breslow depth and tumor stage were obtained from TCGA melanoma dataset; Age, gender and stage were obtained from GEO validation dataset. Univariate and multivariate Cox regression with both IRGs score and clinicopathological features were performed to find out the independent prediction factors significantly associated with survival. To explore whether the IRGs score is helpful in the application of immunotherapy, GSE78220 dataset which includes a melanoma patient cohort treated with anti-PD-1 therapy and pre-treatment RNA sequencing data was analyzed. IRGs score, high- and low-risk groups were generated by the calculation formula and cutoff value described above. The proportions of anti-PD-1 therapy responders and non-responders in low-risk and high-risk groups were obtained.

### Gene set enrichment and pathway analysis (GSEA)

To illustrate the biological functions of the prognostic genes in high-risk and low-risk patient groups, GSEA was performed in java GSEA (verision 3.0) based on the Molecular Signatures Database version 6.2^33^. With the 460 melanoma samples in TCGA dataset, KEGG pathways, biological processes, cellular components, molecular functions associated with high-risk and low-risk groups were identified by using C2 (curated gene sets), C5 (GO gene sets). FDR q value < 0.05, |NES|> 1 were considered statistically significant.

### Validation of CCL8 and DEFB1 expression

Gene expression profiling interactive analysis (GEPIA) is a website server to analyze the RNA sequencing data of tumors and normal samples from the TCGA and the Genotype-Tissue Expression (GTEx) projects (https://gepia.cancer-pku.cn/index.html). Expressions of CCL8 and DEFB1 were plotted with GEPIA in cutaneous melanoma and its subtypes.

### Development and validation of the nomogram

Following multivariate analysis, all independent prognostic predictors including age, stage and IRGs score were used to develop a nomogram. Concordance index, receiver operating characteristic (ROC), area under the curve (AUC) and calibration curves were applied to evaluate the discrimination and accuracy of the nomogram. Decision curve analysis (DCA) was conducted to evaluate the clinical utility of the nomogram and TNM stage through quantifying net benefits against a range of threshold probabilities^34,35^. Finally, the prognostic nomogram was externally validated in the GEO dataset. All analyses were conducted in R software. The packages of R used in this study included “rms”, “foreign”, “survival”, “survivalROC” and "stdca.R". *P* < 0.05 was considered statistically significant unless otherwise noted.

## Results

### Screening of differentially expressed IRGs on melanoma

**Figure 1:**
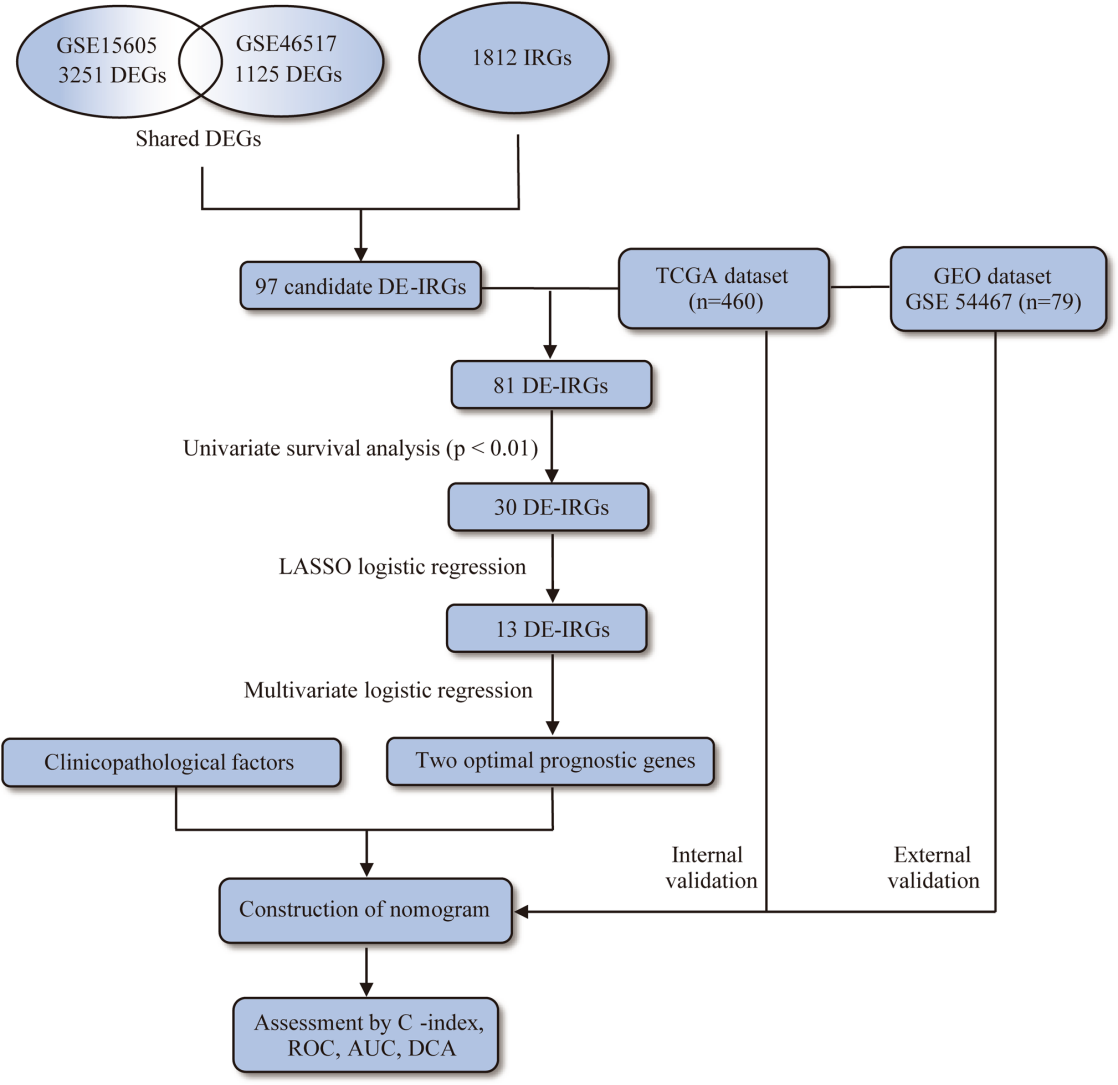
Overall design of the present study. *DEGs* differentially expressed genes, *IRGs* immune-related genes, *DE-IRGs* differentially expressed immune-related genes, *TCGA* the cancer genome atlas, *GEO* gene expression omnibus, *LASSO* least absolute shrinkage and selection operator, *C-index* concordance index, *ROC* receiver operating characteristic, *AUC* area under the curve, *DCA* decision curve analysis.

**Figure 2:**
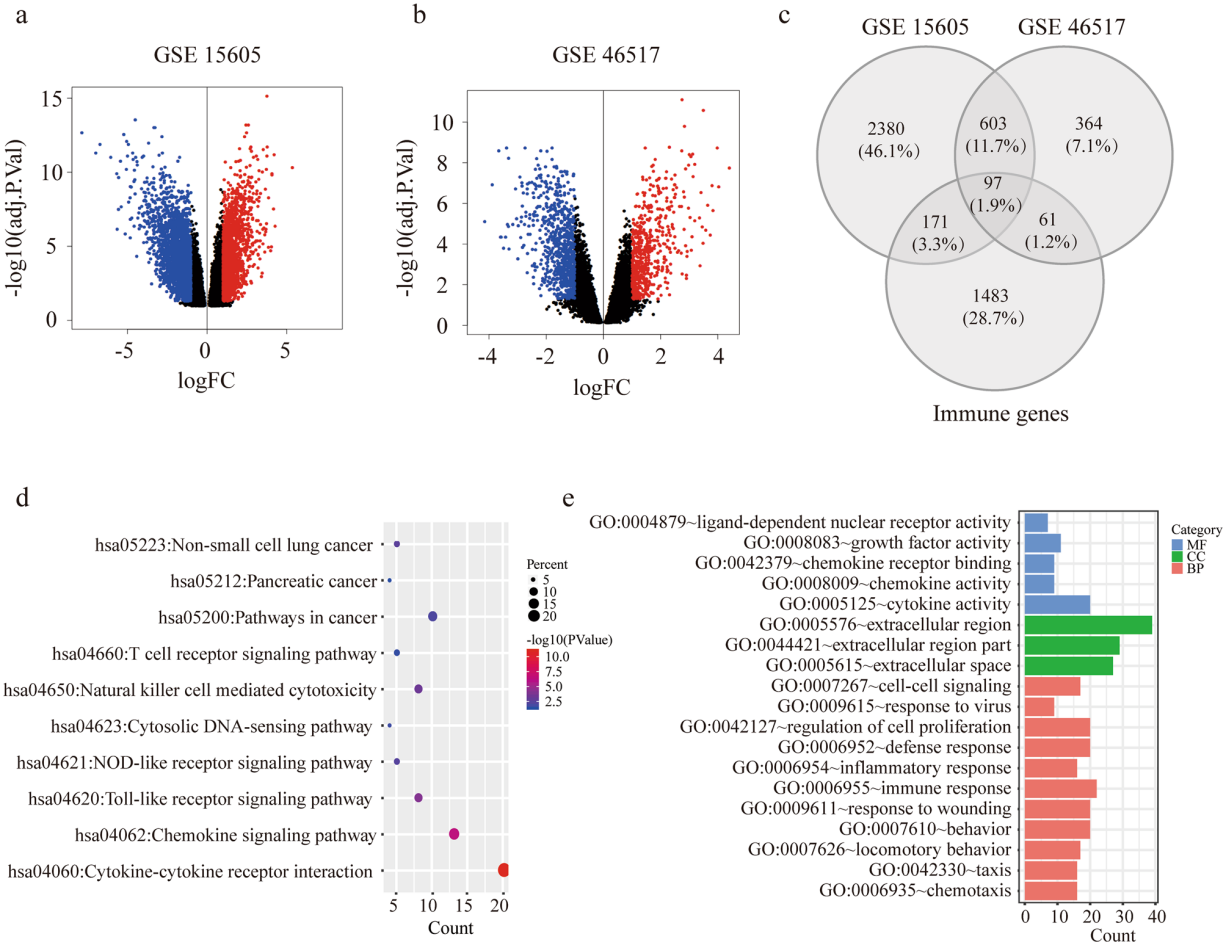
Identification of DE-IRGs and functional enrichment analysis. (**a**,**b**) Volcano plot illustrating differentially expressed genes (DEGs) between melanoma tissue and normal skin in GSE15605 (**a**) and GSE46517 (**b**). (**c**) Venn diagram of the overlapped genes between DEGs and IRGs. (**d**) Enriched Kyoto Encyclopedia of Genes and Genomes (KEGG) pathways of DE-IRGs. (**e**) Enriched Gene Ontology (GO) pathways of DE-IRGs. *MF* molecular funcion, *CC* cell component, *BP* biological process.

The whole workflow for the study was presented in Fig. 1. By comparing expression profiles from melanoma tissue and normal skin in GSE15605 and GSE46517 dataset, 3,251 and 1,125 genes were identified as DEGs respectively with volcano plot analysis (FC ≥ 1, FDR ≤ 0.05) (Fig. 2a, b). A total of 1812 immune-related genes (IRGs) were downloaded from Immport database. Ninety-seven candidate genes, defined as DE-IRGs, were overlapped between DEGs and IRGs and visualized by Venn diagram (Fig. 2c). Finally, 81 DE-IRGs were identified mapped with TCGA (melanoma) dataset and GEO (GSE54467) dataset, where patient survival information was available for downstream prognostic gene identification.

### Functional enrichment and PPI network analysis of DE-IRGs

KEGG and GO enrichment pathway analyses were applied to discover the functions of the 81 DE-IRGs (Fig. 2d,e). The DE-IRGs were remarkably enriched in biological processes related to chemokine signaling pathway and cytokine-cytokine receptor interactions from KEGG analysis. And the extracellular region, immune response and cytokine activity were enriched in the DE-IRGs from GO analysis. These indicated an immune-related, secretary and soluble factor dominant function in DE-IRGs. A PPI network of the 81 DE-IRGs was established, where 76 nodes and 324 interactions was constructed, to identify the interactions between genes (Supplementary figure S1a). The top 15 candidate genes were identified to be significantly involved in the network (Supplementary figure S1b). Module analysis recognized related clustering modules in the PPI network (Supplementary figure S1c). With the DE-IRGs clusters, GO analysis were applied for functional enrichment (Supplementary figure S1d). The results from PPI network and pathway analysis suggested the extracellular region, specifically multiple chemokines and cytokines, were densely connected and enriched in the DE-IRGs.

### Identification of CCL8 and DEFB1 as the independent prognostic DE-IRGs

**Figure 3:**
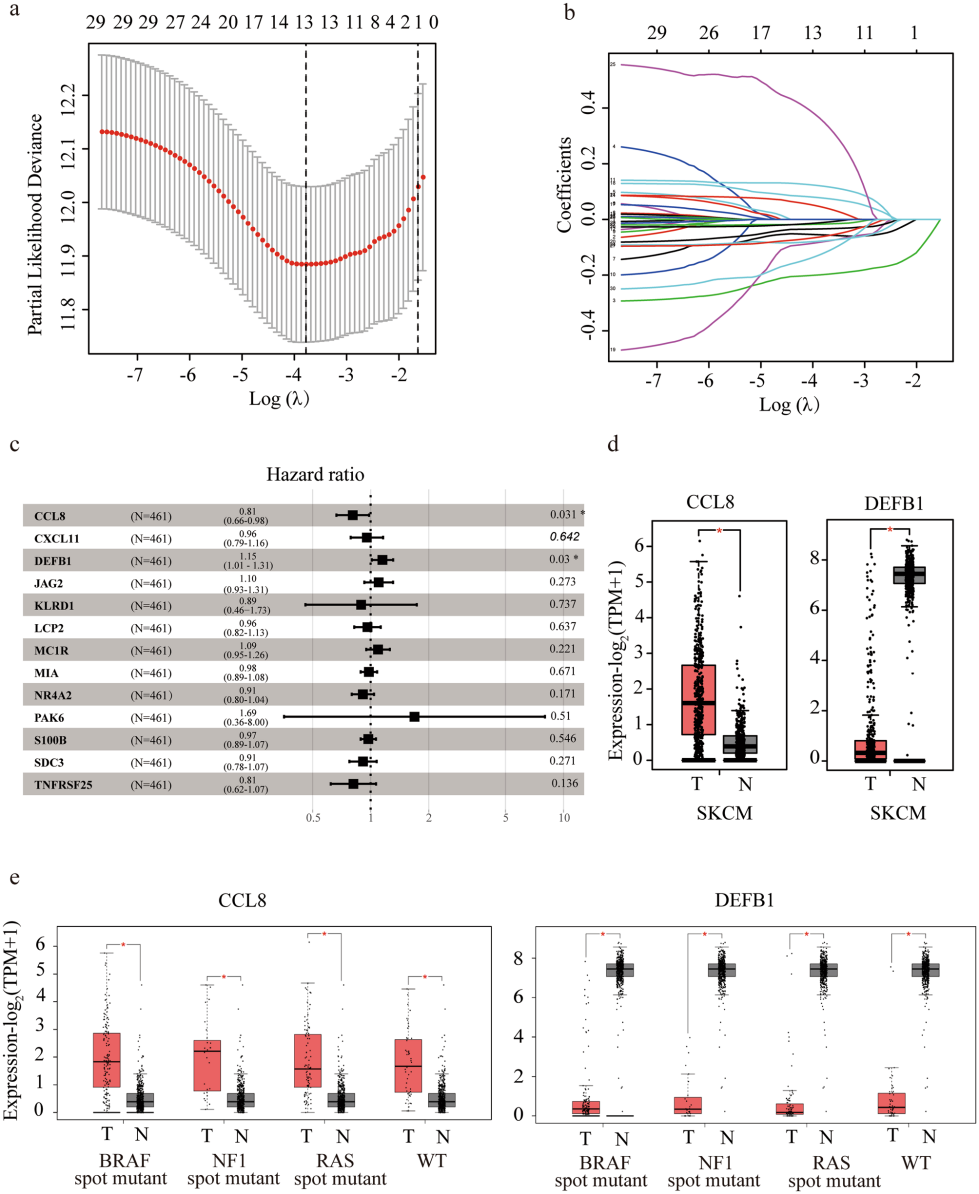
Screening and verification of prognosis-related IRGs. (**a**,**b**) LASSO analysis for selecting the candidate IRGs in TCGA dataset. (**c**) Forest plot by multivariate analysis showing hazard ratio of the candidate IRGs. (**d**,**e**) Boxplots showing expressions of identified IRGs in melanoma tissue and normal skin in general (**d**) or its subtype (**e**) from Gene expression profiling interactive analysis (GEPIA). SKCM: skin cutaneous melanoma; T: tumor; N: normal; WT: wild type.

With the 81 candidate DE-IRGs identified, TCGA melanoma dataset (training) and GEO GSE54467 dataset (validation) were used to recognize the genes associated with survival. Clinical features of these two datasets were summarized in Supplementary table S1. The 81 DE-IRGs in TCGA melanoma dataset were analyzed in univariate Cox analysis, and 30 DE-IRGs were significantly associated with patient survival (*P* < 0.01) (Supplementary table S2). Then, a LASSO logistic regression was applied to avoid collinearity of multiple variables, and 13 DE-IRGs were obtained (Fig. 3a). Coefficient of each gene in TCGA melanoma dataset was illustrated in Fig. 3b. With the 13 DE-IRGs selected, multivariate Cox regression were further performed to figure out the association of gene expression with the patient OS, where CCL8 (HR = 0.81, 95% CI 0.66–0.98, *P* = 0.031) and DEFB1 (HR = 1.15, 95% CI 1.01–1.31, *P* = 0.030) were finally identified to be the independent prognostic genes (Fig. 3c). In addition, the differential expressions of CCL8 and DEFB1 were validated with GEPIA program in all mutation subtypes including BRAF, NF1, RAS mutations and triple wild type (Fig. 3d,e).

### Development and validation of IRGs score model

**Figure 4:**
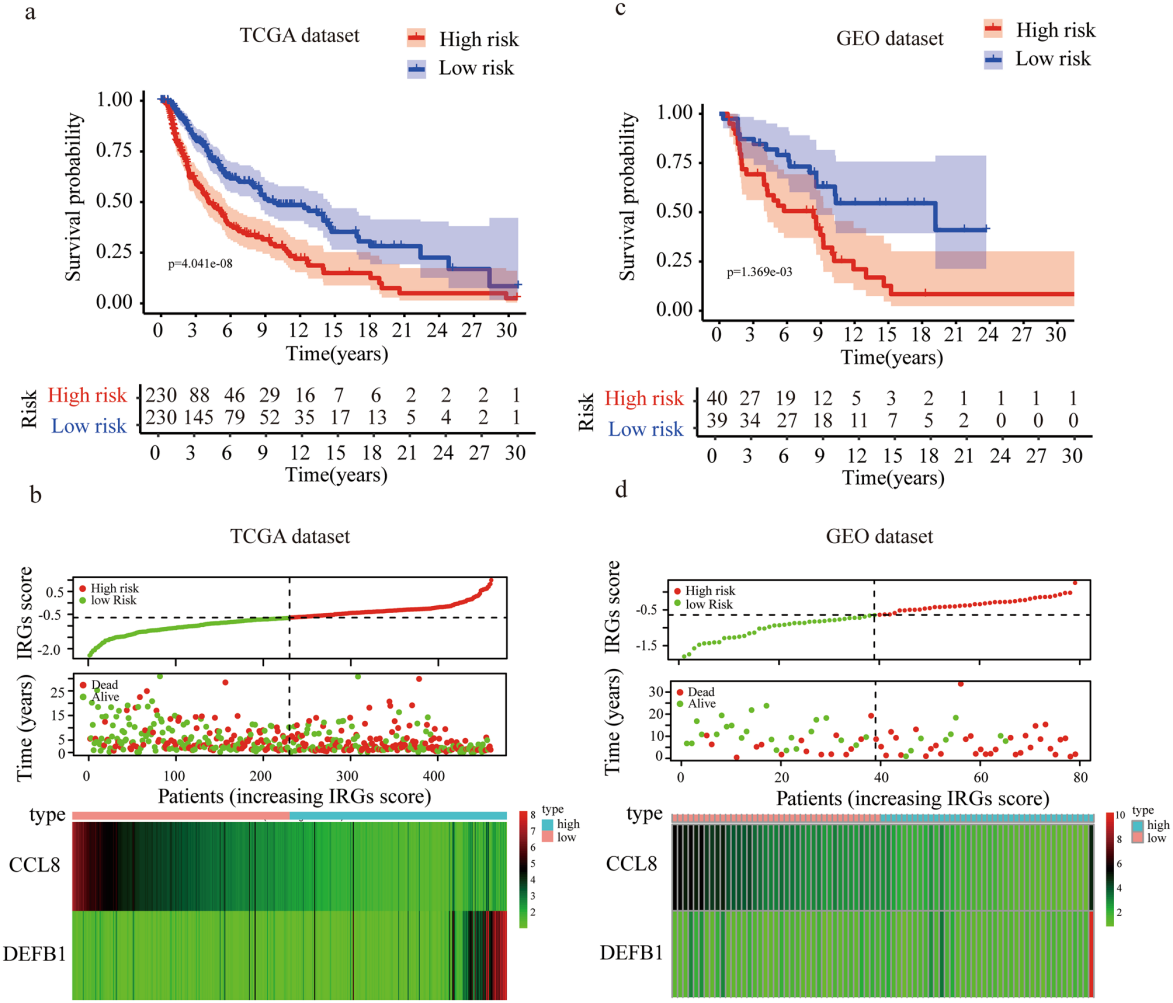
Prognostic analysis of the IRGs signature. (**a**,**c**) Kaplan–Meier survival curves of patients in high versus low risk groups in TCGA (**a**) and GEO datasets (**c**). (**b**,**d**) Distribution of IRGs score and survival days of each patient, and a heatmap of selected IRG expression profiles presented in order of IRGs score in TCGA (**b**) and GEO datasets (**d**).

The independent prognostic genes CCL8 and DEFB1 were chosen to establish a risk score model. From multivariate Cox regression, the coefficients for CCL8 and DEFB1 were − 0.364 and 0.200 respectively. Therefore, the IRG score of each patient was calculated according to the formula: IRGs score = (− 0.364) × (expression value of CCL8) + 0.200 × (expression value of DEFB1). The patients were divided into high- and low-risk groups based on the median risk score (− 0.644) in TCGA melanoma dataset. Kaplan–Meier survival analysis showed that patients in the high-risk group had significantly shorter OS than those in the low-risk group (Fig. 4a). The distribution of the risk score, OS, expressions of CCL8 and DEFB1 were also presented, suggesting that patients with high-risk score had lower expressions of CCL8, higher expressions of DEFB1 and more death cases (Fig. 4b). To confirm the robustness of our model, GEO dataset (GSE54467) which included 79 melanoma patients was applied as the external validation. The patients were divided into high- and low-risk groups according to the same formula and cutoff above. Consistent with the previous results, high-risk patients had significantly worse survival than low-risk patients (Fig. 4c). Expressions of decreased CCL8, increased DEFB1 and more deaths were found in high-risk group than in low-risk group in the GEO validation dataset (Fig. 4d). To further explore the application of IRGs in immunotherapy, we analyzed the pre-treatment mRNA data of 26 melanoma patients who received anti-PD-1 therapy in GSE78220 dataset. According to the IRGs score model described above, 11 and 15 patients were identified as high- and low-risk patients respectively. Surprisingly, 9 (81.8%) high-risk patients responded to the therapy, while only 5 (33.3%) low-risk patients responded, which suggested the high-risk patients were more sensitive to anti-PD-1 treatment than the low-risk group (Supplementary figure S2).

### Gene set enrichment and pathway analysis for DE-IRGs

To investigate the underlying molecular mechanism of the IRGs signature, we conducted GSEA comparing the high-risk group with the low-risk group in 460 melanoma patients from TCGA. There was no GO or KEGG pathway significantly enriched in the high-risk group. However, 946 and 35 pathways in GO and KEGG analysis were identified to be associated with low-risk group, and the top 10 significant terms for each module were summarized in Supplementary table S3. The results demonstrated a major role of antigen presenting cells and T cells in the low-risk group (Supplementary figure S3).

### Development and validation of nomogram based on IRGs and clinicopathological risk factors

**Figure 5:**
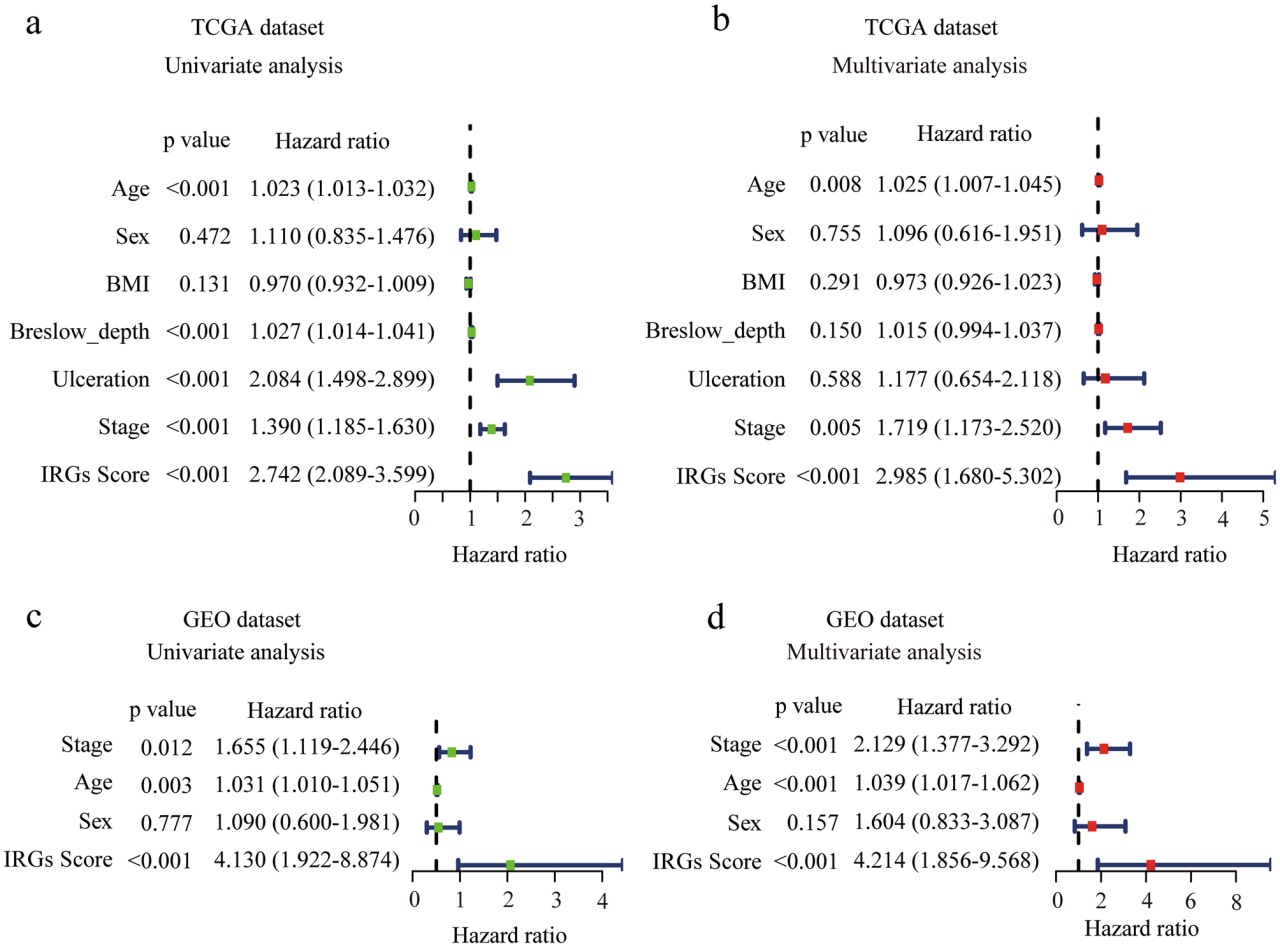
Determination of IRGs score as an independent prognostic factor in melanoma. (**a**,**b**) Forest plot showing the risk of IRGs score and clinical parameters for overall survival by univariate (**a**) and multivariate (**b**) analysis in TCGA dataset. (**c**,**d**) Forest plot showing the risk of IRGs score and clinical parameters for overall survival by univariate (**c**) and univariate (**d**) analysis in GEO dataset. *P* < 0.05 was regarded as statistically significant.

To construct a clinical nomogram that predicts the prognosis of melanoma patients, the clinicopathological factors including age, gender, ulceration, Breslow depth and stage, as well as IRGs score in TCGA melanoma dataset were analyzed with univariate and multivariate Cox analysis (Fig. 5a,b). Importantly, IRGs score was shown to be significantly associated with OS in both with univariate and multivariate analyses. Independent prognostic predictors for melanoma were found to be IRGs score (HR = 2.985, 95%CI 1.680–5.302), age (HR = 1.025, 95%CI 1.007–1.045) and stage (HR = 1.719, 95%CI 1.173–2.520) by multivariate analysis (Fig. 5b). We reached a similar conclusion in GEO validation dataset (IRGs score: HR = 4.214, 95%CI 1.856–9.568, age: HR = 1.039, 95%CI 1.017–1.062, and stage (HR = 2.129, 95%CI 1.377–3.292) (Fig. 5c,d).

**Figure 6:**
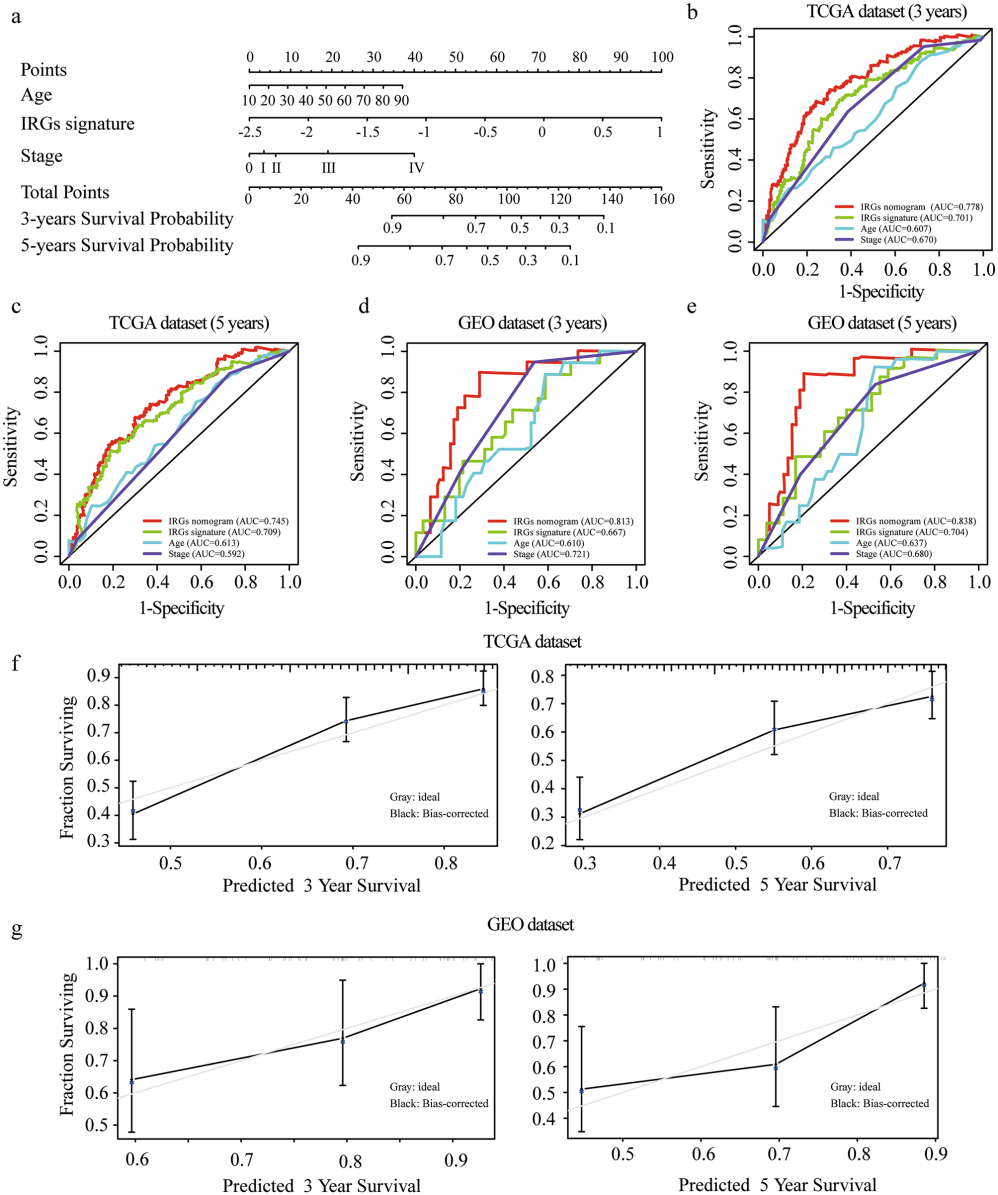
Developing and validating a nomogram based on the IRGs risk score model. (**a**) A prognostic nomogram through combining the IRGs score and clinical parameters. (**b**,**e**) ROC curves of IRGs nomogram compared with stage, age or IRGs score alone in TCGA 3-year (**b**) and 5-year (**c**) prediction and GEO 3-year (**d**) and 5-year (**e**) prediction. (**f**,**g**) Calibration curves for TCGA (**f**) and GEO (**g**) dataset. The grey line represents a perfect prediction, and the black line describes the predictive performance of the nomogram, where the fitness of the black line to the grey line indicates a good prediction by the model.

**Figure 7:**
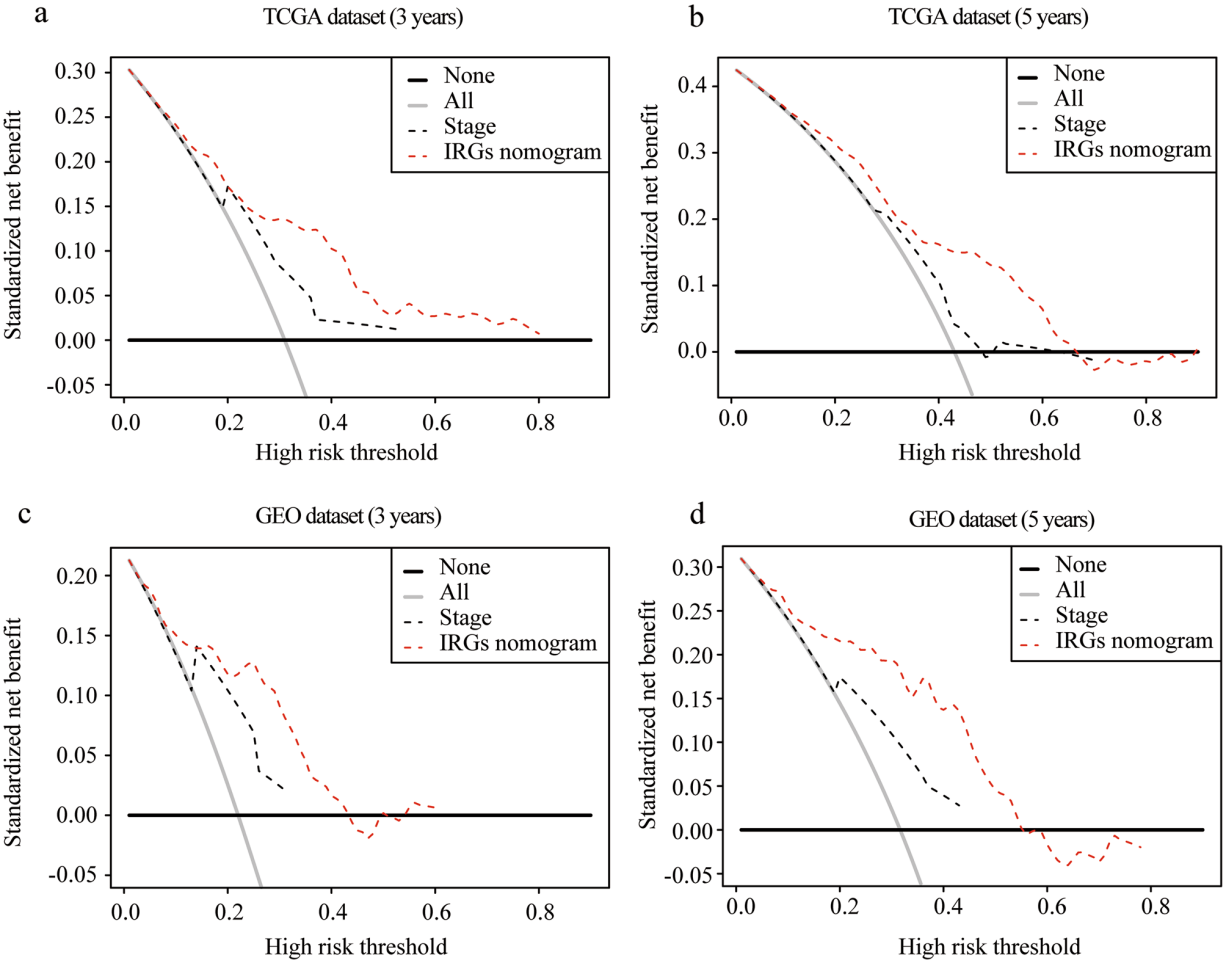
Model comparison and clinical usefulness of IRGs nomogram. Decision curve analysis of the nomogram model in TCGA 3-year (**a**) and 5-year (**b**) prediction and GEO 3-year (**c**) and 5-year (**d**) prediction. The clinical usefulness of IRGs nomogram model and the stage evaluation system were compared to treating none or all of the patients. The higher the net benefit, the better the evaluation model was. In both training and validation sets, using the IRGs nomogram to predict patient prognosis added more benefit than treating none or all patients, and was more beneficial compared to using the stage evaluation to predict.

With the combination of age, stage and IRGs score, we established an IRGs nomogram based on TCGA melanoma dataset to predict the individual risk of 3-year or 5-year survival (Fig. 6a). The concordance index of the nomogram was 0.705. Moreover, ROC analyses suggested that the nomogram achieved a superior 3-year prediction efficacy with an AUC of 0.778 compared to other models such as IRGs signature (0.701), age (0.607), and tumor stage (0.670), and a better 5-year prediction efficacy with an AUC of 0.745 compared to IRGs signature (0.709), age (0.613), and tumor stage (0.592) (Fig. 6b,c). Also, calibration curves indicated excellent agreement between the nomogram prediction and actual observation in terms of the 3-year and 5-year survival rates in the TCGA melanoma dataset (Fig. 6f). In addition, the nomogram of GEO validation dataset reached a concordance index of 0.715. ROC analyses in the validation dataset demonstrated that the nomogram generated an AUC of 0.813 higher than that in IRGs signature (0.667), age (0.610), and tumor stage (0.721) in 3-year prediction, and an AUC of 0.838 in the nomogram higher than that in IRGs signature (0.704), age (0.637), and tumor stage (0.680) in 5-year prediction (Fig. 6d,e). Calibration curves also showed a satisfactory goodness-of-fit in GEO dataset (Fig. 6g). Decision Curve Analysis (DCA) has been used to assess the clinical value of models which integrates the preferences of the patients into analysis. DCAs results for the nomogram and the stage model in 3-year and 5-year survival predictions were presented in Fig. 7, showing that melanoma prognostic prediction based on the nomogram added more net benefit than the “treat all”, “treat none” strategies and the stage model in both TCGA and GEO datasets.

## Discussion

Melanoma is the most invasive type of skin cancer with a challenge to identify prognostic biomarkers. As a cancer largely regulated by immune system, the development of immune-related biomarkers would be of much value^36–38^. And prediction model for prognosis based on multiple markers including immune genes would potentially help with selecting the optimal therapy in the clinic.

In this study, we sorted out the most influential immune-related genes affecting patient survival with public database and bioinformatical method. Combining several clinical features, we generated a nomogram model that was capable of predicting patient outcomes. Notably, a satisfying AUC was obtained with patient age, stage and expressions of only two genes, which means that in our model, a relatively accurate survival prediction for prognosis can be achieved with a handful of accessible parameters. Further, the comparison between nomogram and other models demonstrated that, our nomogram, which included staging information, had a significantly higher efficiency than staging system alone, with AUC raised from 0.670 to 0.778 in 3-year prediction and from 0.592 to 0.745 in 5-year prediction. This suggests that although tumor stage is a traditional indicator when predicting prognosis, the additional factors in our nomogram, IRGs signatures consisting CCL8 and DEFB1, and the age of patients, are also worth considering.

CCL8 was shown to be negatively correlated with high-risk status in our study. In present literatures, CCL8 is a cytokine that promotes the metastasis in kinds of tumors, including breast cancer, lung cancer and esophageal squamous cell carcinoma^39–41^. The role of CCL8 is controversial when it comes to melanoma. Tamas Barbai et al. found an increased migration of melanoma cell lines with CCL8 added as a chemoattractant, whereas Kiyokazu Hiwatashi et al. demonstrated that CCL8 suppressed metastatic ability of B16F10 melanoma cells^42,43^. Our results, however, showed that higher expression of CCL8 indicated better survival in patients, which was confirmed by external validation. This is in favor of the point that CCL8 in melanoma might play a protective role overall, of which the mechanism warrants further investigation.

DEFB1 is a peptide with multiple immune-related functions and is thought to be a tumor suppressor^44,45^. It was found to be downregulated in renal, prostate and colorectal cancers^46,47^. Our study demonstrated for the first time, an aberrant expression of DEFB1 in melanoma compared to normal skin. However, little was known about its function in melanoma, except for that Lara Fernandez et al. reported that genetic variations of DEFB1 might be correlated with the risk for melanoma, but they did not perform confirmatory studies on this conclusion^48^. In this study, we showed that the higher DEFB1 expression indicated unfavorable prognosis, which would be informative in clinical evaluation for patients. On the other hand, though identified as a tumor suppressor gene by previous research, DEFB1 might play a different role in melanoma than other types of cancer, which could potentially fuel mechanistic research on its unfavorable role in melanoma. Taken together, DEFB1 is indicative of patient survival and is a potential biomarker in melanoma.

We attempted to apply our IRGs score model to predict patient response to anti-PD-1 therapy, and it showed differential response rates in low- and high-risk group patients, indicating that the high-risk group was more sensitive to anti-PD-1 treatment, while the low-risk group was tend to be resistant. However, there were only 26 patients in this cohort, although the difference is statistically significant, larger scale validation is required to make a convincing conclusion.

Admittedly, there are a few limitations in our analysis. Firstly, our analysis is based on expressions at messenger RNA level, without regard to protein level expressions or posttranscriptional modifications, which also have important biological effects. Further, we applied DEGs between melanomas and normal controls in predicting patient survivals, however, we might have ignored some genes with critical prognostic value that do not necessarily differ between melanomas and normal controls. Lastly, there is a lack of validation by more melanoma cohorts, which is limited by the data availability.

## Conclusions

In conclusion, we have constructed a predictive model which combined immune-related genes with clinical characteristics for the first time, to estimate melanoma patient survivals and therefore help with decision making in the treatment.

## Supplementary information

Supplementary information

## Data Availability

The GEO datasets analyzed during the current study are available in the Gene Expression Omnibus repository, https://www.ncbi.nlm.nih.gov/geo/query/acc.cgi?acc=GSE15605, https://www.ncbi.nlm.nih.gov/geo/query/acc.cgi?acc=GSE46517, https://www.ncbi.nlm.nih.gov/geo/query/acc.cgi?acc=GSE54467. The TCGA datasets analyzed during the current study are available in The Cancer Genome Atlas repository, https://xenabrowser.net/datapages.

## Abbreviations

PD-1: Programmed death-1
CLTA-4: Cytotoxic T-lymphocyte antigen 4
GEO: Gene expression omnibus
TCGA: The cancer genome atlas
DEGs: Differentially expressed genes
IRGs: Immune-related genes
DE-IRGs: Differentially expressed immune-related genes
KEGG: Kyoto encyclopedia of genes and genomes
GO: Gene ontology
PPI: Protein-protein interactions
LASSO: Least absolute shrinkage and selection operator
OS: Overall survival
GEPIA: Gene expression profiling interactive analysis
ROC: Receiver operating characteristic
AUC: Area under the curve
DCA: Decision curve analysis
HR: Hazard ratio
CI: Confidence interval
GSEA: Gene set enrichment analysis
