# Supplementary material for: A novel predictive model incorporating immune-related gene signatures for overall survival in melanoma patients
Source: Sci Rep. 2020 Jul 27;10:12462. doi: 10.1038/s41598-020-69330-2 (PMC7385638; doi:10.1038/s41598-020-69330-2)
Supplement: Supplementary file 1 — Supplementary information [file 41598_2020_69330_MOESM1_ESM.pdf]

A novel predictive model incorporating immune-related gene signatures for overall survival in melanoma patients

Mengting Liao[1, 2, #], Furong Zeng[2, #], Yao Li[2], Qian Gao[2], Mingzhu Yin[2], Guangtong Deng[2, *], Xiang Chen[2, *]

[1]Health Management Center, Xiangya Hospital, Central South University, Changsha, 410008, China

[2]The Department of Dermatology, Xiangya Hospital, Central South University, China; Hunan Key Laboratory of Skin Cancer and Psoriasis, China; Hunan Engineering Research Center of Skin Health and Disease, 410008, China

[#]These authors contributed equally to this work.

[*]Co-corresponding author

Corresponding authors: Xiang Chen, Guangtong Deng, Dermatology department of Xiangya hospital, Central South University, Xiangya Road 87, Changsha 410008, China. E-mail: chenxiangck@126.com, dengguangtong@outlook.com, TEL: +8613723890471

Supplementary table S1. Summary of clinical features from TCGA dataset and GEO dataset.

	TCGA dataset	GEO dataset GSE54467
Total number of patients	460	79
Gender (n, %)		
Male	286 (62.3%)	50 (63.3%)
Female	174 (37.7%)	29 (36.7%)
Age (mean ± SD, yr)	58.1 ± 15.7	56.2 ± 15.1
Stage (n, %)		
0	6 (1.3%)	0 (0%)
I	77 (16.7%)	29 (36.7%)
II	139 (30.2%)	29 (36.7%)
III	169 (36.7%)	20 (25.3%)
IV	23 (5.0%)	0 (0%)
Unknown	46 (10.0%)	1 (1.3%)
OS (mean ± SD, m)	61.4 ± 64.5	97.9 ± 47.3

SD: standard deviation; OS: overall survival; yr: years; m: month.

Supplementary table S2. Univariate Cox regression analysis of DE-IRGs and overall survival in TCGA melanoma cohort.

Gene ID	HR	HR.95L	HR.95H	Pvalue
ACTA1	1.205215	0.962924	1.508472	0.103094
AGTR1	0.963104	0.720639	1.287148	0.799451
AHNAK	0.978846	0.854795	1.120899	0.757138
AZGP1	0.964432	0.892619	1.042022	0.358966
BMP1	1.281857	1.030825	1.59402	0.02555
BMP2	0.908111	0.769628	1.071512	0.253545
**BST2**	**0.845844**	**0.785682**	**0.910613**	**8.69E-06**
CCL18	0.988209	0.913182	1.069401	0.76844
CCL27	0.562083	7.35E-05	4297.444	0.899513
**CCL5**	**0.84383**	**0.788496**	**0.903047**	**9.25E-07**
**CCL8**	**0.694869**	**0.617175**	**0.782343**	**1.77E-09**
CD320	0.946971	0.790397	1.134561	0.554595
**CD8A**	**0.800297**	**0.733799**	**0.872823**	**4.82E-07**
CTSB	0.942835	0.831496	1.069083	0.358575
**CTSS**	**0.806862**	**0.734519**	**0.88633**	**7.55E-06**
**CXCL10**	**0.836568**	**0.786594**	**0.889716**	**1.36E-08**
**CXCL11**	**0.731872**	**0.656038**	**0.816473**	**2.23E-08**
**CXCL13**	**0.835448**	**0.77697**	**0.898327**	**1.20E-06**
CXCL2	0.840081	0.694822	1.015709	0.072011
**CXCL9**	**0.853731**	**0.806261**	**0.903996**	**6.03E-08**
**CXCR3**	**0.768704**	**0.687611**	**0.859362**	**3.75E-06**
**DEFB1**	**1.237586**	**1.117333**	**1.370782**	**4.37E-05**
DES	1.084452	0.983034	1.196333	0.105578
EDNRB	1.093113	0.9953	1.200537	0.062677
EGFR	1.163622	1.005983	1.345963	0.041323
**EIF2AK2**	**0.718473**	**0.59084**	**0.873677**	**0.000922**
ELN	0.964368	0.865749	1.074222	0.509781
F2RL1	0.952806	0.80008	1.134684	0.587556
FGFR3	1.192654	1.034791	1.374599	0.015013
**GAL**	**1.182914**	**1.068358**	**1.309754**	**0.001228**
GDF11	0.955031	0.833797	1.093893	0.506506
GDF15	0.992737	0.936328	1.052545	0.807062
GREM1	1.042412	0.816918	1.330149	0.738387
**GZMB**	**0.811756**	**0.741747**	**0.888372**	**5.84E-06**
**ICAM1**	**0.826023**	**0.745179**	**0.915638**	**0.000276**
IL11RA	0.950107	0.810063	1.11436	0.5293
**IL18**	**0.746608**	**0.650715**	**0.856633**	**3.10E-05**
IL1R2	0.757387	0.567386	1.011014	0.059344
IL1RN	1.014443	0.88837	1.158407	0.832275

IL6	0.880142	0.740679	1.045864	0.146922
INHBB	0.914877	0.782879	1.069132	0.2631
**ISG15**	**0.8724**	**0.802568**	**0.948309**	**0.001342**
ISG20	0.863044	0.762648	0.976655	0.019578
**JAG2**	**1.324683**	**1.124891**	**1.559959**	**0.000749**
**KLRD1**	**0.333149**	**0.212289**	**0.522818**	**1.75E-06**
**LCP2**	**0.729765**	**0.645119**	**0.825517**	**5.49E-07**
**MC1R**	**1.205559**	**1.054378**	**1.378418**	**0.006248**
**MIA**	**0.906551**	**0.841357**	**0.976796**	**0.009979**
MICB	0.908448	0.78885	1.046178	0.182479
**MMP9**	**0.917133**	**0.859242**	**0.978924**	**0.009315**
MX2	0.922901	0.830311	1.025815	0.136896
NR3C2	0.700433	0.529061	0.927315	0.01288
**NR4A2**	**0.834581**	**0.734317**	**0.948536**	**0.005622**
OGN	1.032496	0.882146	1.20847	0.690435
**PAK6**	**9.68596**	**2.93539**	**31.96094**	**0.000193**
PDGFRL	0.943659	0.824095	1.08057	0.401499
PIK3CD	1.0763	0.95024	1.219084	0.247314
PLA2G2A	0.902687	0.826868	0.985458	0.022183
PLAUR	0.914668	0.820259	1.019943	0.108561
PLXNC1	1.039859	0.949649	1.138638	0.39858
PTGER3	1.040868	0.721007	1.502628	0.830688
RORA	0.843506	0.599733	1.186365	0.328097
RXRA	1.317597	1.053713	1.647565	0.015572
RXRG	0.923294	0.862965	0.987839	0.020621
S100A1	0.960184	0.905788	1.017846	0.172099
**S100B**	**0.916157**	**0.858436**	**0.977758**	**0.008354**
SCG2	0.895738	0.811701	0.988475	0.028481
SDC1	1.062829	0.968594	1.166232	0.198326
**SDC3**	**0.812366**	**0.707665**	**0.932557**	**0.003159**
SDC4	0.846877	0.728228	0.984857	0.03092
SEMA3F	1.0866	0.907579	1.300933	0.365887
SPP1	0.935421	0.881322	0.992841	0.028067
**STAT1**	**0.752251**	**0.680443**	**0.831637**	**2.67E-08**
TGFA	0.97803	0.882713	1.083638	0.671106
TNFRSF10B	0.983715	0.8716	1.110252	0.790282
**TNFRSF21**	**0.875259**	**0.792846**	**0.966238**	**0.008274**
**TNFRSF25**	**0.672855**	**0.529844**	**0.854466**	**0.001154**
TPM2	0.973666	0.860197	1.102102	0.672919
VAV3	0.864364	0.765661	0.975791	0.018468
VDR	0.84911	0.721185	0.999728	0.04962
VIPR1	1.35603	0.929297	1.978718	0.114188

Supplementary Table S3. Gene set enrichment analysis in high- and low-risk group patients from TCGA melanoma dataset

NAME	SIZE	ES	NES	NOM p-val	FDR q-val
Enrichment for low-risk patients in GO analysis					
**GO Biological process**					
GO_FC_EPSILON_RECEPTOR_SIGNALING_PATHWAY	124	-0.57328	-2.41404	0	0.0079535
GO_POSITIVE_REGULATION_OF_VIRAL_GENOME_ REPLICATION	30	-0.62815	-2.41406	0	0.0089477
GO_INNATE_IMMUNE_RESPONSE_ACTIVATING_CELL_ SURFACE_RECEPTOR_SIGNALING_PATHWAY	105	-0.67163	-2.44447	0	0.0094596
GO_ANTIGEN_PROCESSING_AND_PRESENTATION_ OF_PEPTIDE_ANTIGEN_VIA_MHC_CLASS_I	84	-0.68191	-2.36016	0	0.0094726
GO_POSITIVE_REGULATION_OF_PROTEIN_ MODIFICATION_BY_SMALL_PROTEIN_CONJUGATION_ OR_REMOVAL	194	-0.40358	-2.3286	0	0.009745
GO_RESPONSE_TO_VIRUS	244	-0.73905	-2.33487	0	0.010159
GO_REGULATION_OF_RNA_STABILITY	139	-0.38445	-2.36743	0	0.0103337
GO_NIK_NF_KAPPAB_SIGNALING	83	-0.53294	-2.33226	0	0.0103947
GO_ANTIGEN_PROCESSING_AND_PRESENTATION	189	-0.58555	-2.37056	0	0.0104191
GO_FC_GAMMA_RECEPTOR_SIGNALING_PATHWAY	73	-0.73648	-2.33939	0	0.0104306
**GO Cellular component**					
GO_COP9_SIGNALOSOME	34	-0.68723	-2.36893	0	0.0046815
GO_AUTOPHAGOSOME	76	-0.47928	-2.23899	0	0.0092122
GO_PHAGOCYTIC_VESICLE	77	-0.70556	-2.25725	0	0.0123529
GO_PHAGOCYTIC_VESICLE_MEMBRANE	50	-0.65431	-2.1781	0	0.0173421
GO_CYTOPLASMIC_MRNA_PROCESSING_BODY	70	-0.47407	-2.10441	0	0.0220603
GO_PODOSOME	23	-0.78744	-2.09548	0	0.0222957
GO_SIDE_OF_MEMBRANE	383	-0.67507	-2.07996	0	0.0230799
GO_ENDOLYSOSOME	16	-0.84275	-2.0866	0	0.0233054
GO_ENDOSOMAL_PART	395	-0.42569	-2.11635	0	0.0239201
GO_LAMELLIPODIUM	170	-0.49884	-2.02708	0	0.0356465
**GO Molecular function**					
GO_DOUBLE_STRANDED_RNA_BINDING	62	-0.66166	-2.36782	0	0.004743
GO_SIGNALING_ADAPTOR_ACTIVITY	74	-0.68347	-2.09203	0	0.0188616
GO_CYTOKINE_RECEPTOR_BINDING	265	-0.6751	-2.06301	0	0.0195865

GO_GUANYL_NUCLEOTIDE_BINDING	360	-0.47455	-2.07142	0	0.0201563
GO_PROTEIN_PHOSPHATASE_BINDING	117	-0.45785	-2.03275	0	0.0211438
GO_SH2_DOMAIN_BINDING	29	-0.79359	-2.09205	0	0.0212193
GO_PROTEIN_N_TERMINUS_BINDING	102	-0.42595	-2.23208	0	0.0212831
GO_PHOSPHATIDYLINOSITOL_3_4_5_TRISPHOSPHATE_BINDING	34	-0.61773	-2.01739	0.001887	0.0217976
GO_SINGLE_STRANDED_RNA_BINDING	67	-0.47169	-2.03464	0	0.0224394
GO_SH3_SH2_ADAPTOR_ACTIVITY	52	-0.75257	-2.09834	0	0.0225416
GO_DOUBLE_STRANDED_RNA_BINDING	62	-0.66166	-2.36782	0	0.004743
**KEGG**					
KEGG_RIG_I_LIKE_RECEPTOR_SIGNALING_PATHWAY	71	-0.6119	-1.9624	0	0.02136
KEGG_NOD_LIKE_RECEPTOR_SIGNALING_PATHWAY	62	-0.7398	-1.9153	0	0.02149
KEGG_RENAL_CELL_CARCINOMA	70	-0.4416	-1.9654	0	0.02261
KEGG_VIRAL_MYOCARDITIS	50	-0.6642	-1.9158	0	0.02268
KEGG_LEUKOCYTE_TRANSENDOTHELIAL_MIGRATION	115	-0.6373	-1.9485	0	0.02273
KEGG_TOLL_LIKE_RECEPTOR_SIGNALING_PATHWAY	102	-0.755	-2.1685	0	0.02311
KEGG_NON_SMALL_CELL_LUNG_CANCER	54	-0.4858	-1.8887	0.0038	0.02318
KEGG_PATHOGENIC_ESCHERICHIA_COLI_INFECTION	53	-0.5274	-1.892	0	0.02369
KEGG_LEISHMANIA_INFECTION	58	-0.7953	-1.9169	0	0.02382
KEGG_T_CELL_RECEPTOR_SIGNALING_PATHWAY	108	-0.711	-1.9727	0	0.02409

Supplementary figure S1. Protein-protein interactions (PPI) network analysis of DE-IRGs.

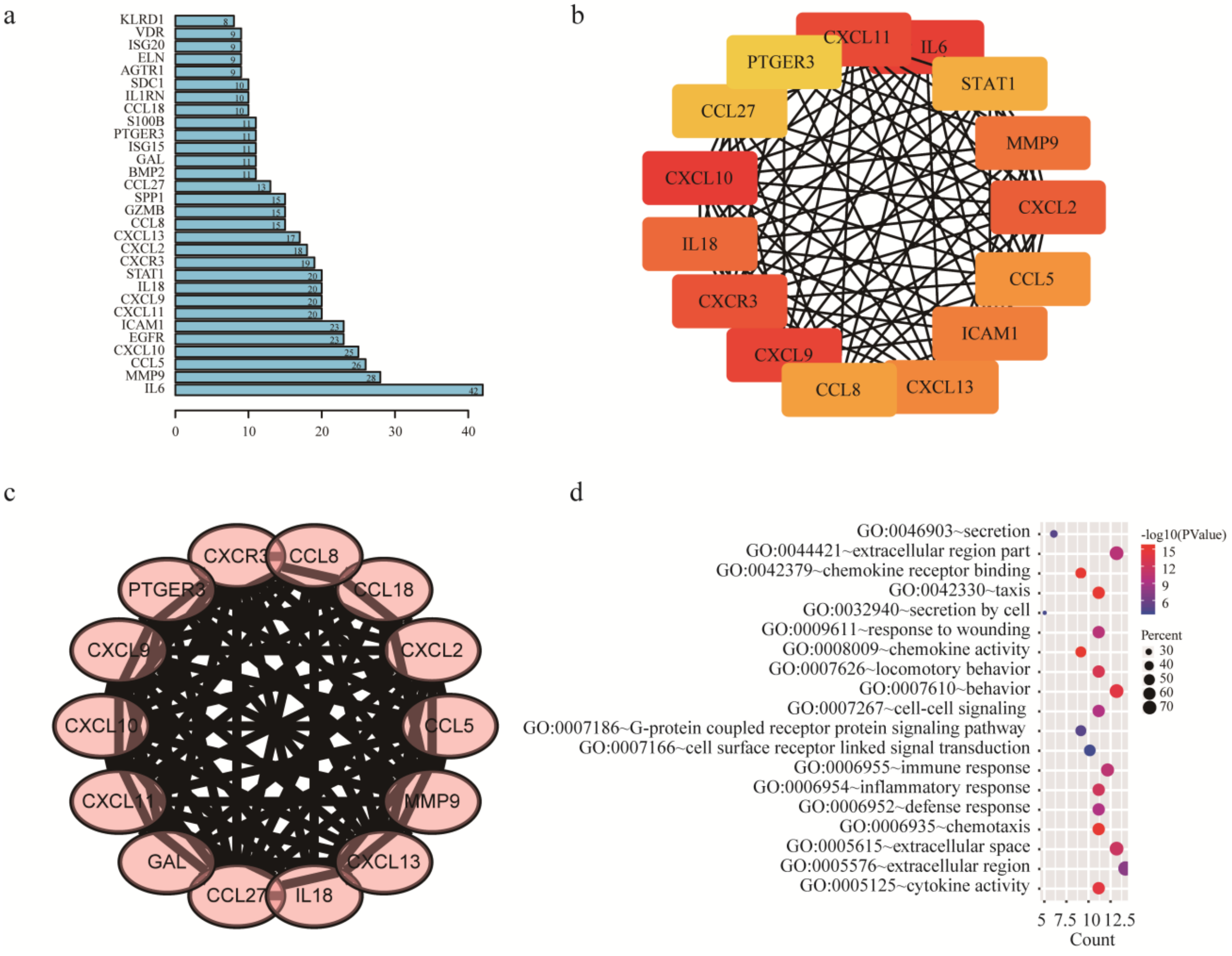

(a) Bar plot showing the representative nodes enriched for gene interaction identification. (b) The network of the top 15 candidate hub genes. (c) Clustering module in the PPI network. (d) The most significant functional enrichment in the clustering module.

Supplementary figure S2. Immunotherapy response in low- and high-risk patients by IRGs score.

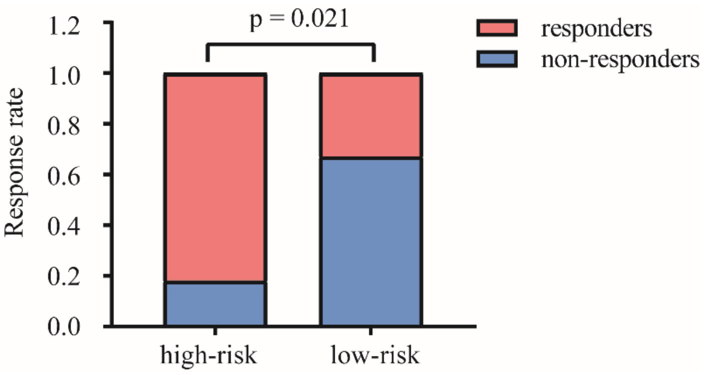

Bar graph showing the differential response rate to anti-PD-1 therapy in low- and high-risk patients.

Supplementary figure S3. Gene set enrichment and pathway analysis (GSEA).

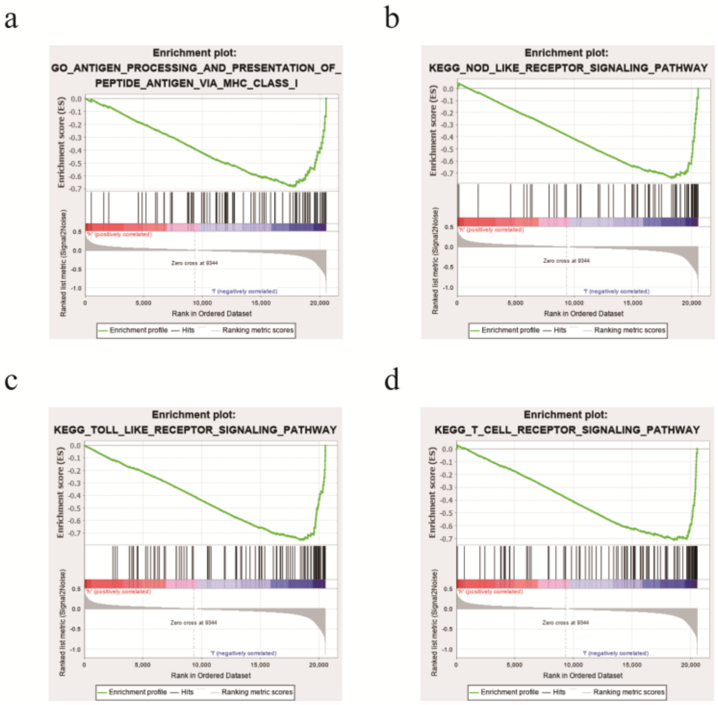

Representative GSEA plots for GO and KEGG pathway enrichment in low-risk patients. Signaling pathways related to antigen presenting cells and T cells were enriched.
